# Multi-omics advances in sepsis-associated encephalopathy: from pathogenesis to clinical translation

**DOI:** 10.3389/fimmu.2026.1941590

**Published:** 2026-10-07

**Authors:** Linlin Gao, Xuehua Liu

**Affiliations:** Department of General Practice, Tianjin Union Medical Center, The First Affiliated Hospital of Nankai University, Tianjin, China

**Keywords:** biomarkers, multi-omics integration, neuroinflammation, precision medicine, sepsis-associated encephalopathy

## Abstract

Sepsis-associated encephalopathy (SAE) represents the most prevalent neurological complication arising from sepsis, affecting approximately 70% of patients with sepsis and associated with mortality rates reaching up to 70%. Survivors frequently experience persistent cognitive deficits; however, the field currently lacks specific diagnostic biomarkers and targeted therapeutic interventions, with diagnosis primarily established through exclusion criteria. This review critically examines multi-omic investigations of SAE, evaluating whether convergent evidence across genomic, transcriptomic, proteomic, and metabolomic domains elucidates shared pathophysiological mechanisms and identifying prevailing translational challenges. The synthesis of evidence is structured around three interrelated thematic domains. First, multi-omic analyses have elucidated the initial triggers of neuroinflammation: genomic studies implicate Piezo1-mediated oligodendrocyte ferroptosis and adenosine-to-inosine (A-to-I) RNA editing, while proteomic data corroborate activation of the reactive oxygen species (ROS)/thioredoxin-interacting protein (TXNIP)/NLRP3 inflammasome axis, collectively indicating that peripheral inflammatory cues converge upon central innate immune pathways. Second, transcriptomic and proteomic findings converge on microglial polarization as a pivotal mediator in SAE progression: single-cell sequencing reveals C1q-dependent synaptic pruning, S100A9 protein expression promotes pro-inflammatory microglial polarization, and metabolomic profiles demonstrate that cyclophilin D (CypD)-regulated redox imbalances modulate the magnitude of glial inflammatory responses. Third, integrative analyses delineate neuronal injury pathways: the NAT10 (N-acetyltransferase 10)/GABA(B) receptor 1 axis is implicated in inhibitory synaptic dysfunction through coordinated transcriptomic and proteomic alterations, while elevated levels of 4-hydroxyphenylacetic acid correlate with impaired consciousness, suggesting its potential utility as a prognostic biomarker. Across these domains—initiation, mediation, and injury—multi-omic integration further extends the conceptual framework to encompass systemic regulation via the microbiota–gut–brain axis. Notwithstanding these advances, extant evidence is limited by predominantly cross-sectional study designs, reliance on single-animal models, and a paucity of rigorous clinical validation. To address these limitations, we propose a three-tiered cascade model—comprising Inflammation Initiation, Glial Mediation, and Neuronal Injury—that synthesizes current findings into a coherent, testable framework. Furthermore, we delineate a methodological roadmap emphasizing longitudinal sampling, multi-center validation, causal inference methodologies, and artificial intelligence-driven data integration. This review endeavors not only to consolidate existing knowledge but also to establish an actionable translational paradigm aimed at advancing SAE research from descriptive characterization toward precision diagnostics and mechanism-based therapeutic strategies.

## 1 Introduction

A previously healthy 65-year-old individual was admitted with community-acquired pneumonia. By the second day of hospitalization, the patient developed septic shock. On the third day, the clinical picture evolved to include agitation, disorientation, and progressive somnolence. Neuroimaging via computed tomography of the head revealed no abnormalities, and cerebrospinal fluid analysis was unremarkable. Attempts to improve mental status through sedation interruption were unsuccessful. The clinical team faced a familiar diagnostic challenge: the presentation was strongly indicative of sepsis-associated encephalopathy (SAE), yet this diagnosis remains one of exclusion. Currently, there is no definitive diagnostic test, validated biomarker, or targeted therapeutic intervention available. This scenario exemplifies the clinical reality of SAE as of 2026.

SAE is characterized as a diffuse cerebral dysfunction resulting from severe sepsis in the absence of direct central nervous system infection (1). Its clinical manifestations range from inattention and agitation to stupor and coma (2). SAE affects approximately 70% of patients with sepsis and is associated with a mortality rate approaching 70% (3, 4). Among survivors, persistent neurocognitive impairments may endure for years, significantly diminishing quality of life and imposing substantial socioeconomic burdens (5). Despite its high prevalence and severity, SAE remains a poorly understood clinical entity: its pathophysiological mechanisms are incompletely characterized, no definitive biomarkers have been identified, and treatment remains limited to symptomatic management (6).

The intractability of SAE can be attributed to its inherent complexity. Its pathophysiology involves a dynamic interplay among neuroinflammation, disruption of the blood-brain barrier, oxidative stress, mitochondrial dysfunction, neurotransmitter imbalances, and widespread desynchronization of brain networks (6). However, most mechanistic insights have been derived from animal models that investigate isolated pathways, limiting their translational applicability to human SAE, which is characterized by variable onset, fluctuating clinical course, and heterogeneous presentations. Importantly, SAE exhibits clinical heterogeneity: some patients experience transient and fully reversible delirium, whereas others rapidly progress to deep coma or death. This variability suggests that SAE may not represent a singular disease entity but rather a common final pathway resulting from multiple distinct pathophysiological processes. If this hypothesis holds true, the pursuit of a universal biomarker may be fundamentally misguided (7, 8).

Over the past two decades, biomarker research has largely substantiated this concern. Candidate biomarkers such as neuron-specific enolase (NSE), S100β, and interleukin-6 have been extensively studied; however, none have been incorporated into routine clinical practice (9). These markers lack specificity for SAE, as their levels are elevated in sepsis without encephalopathy, traumatic brain injury, and stroke, and they fail to reliably differentiate SAE from sedative effects, metabolic encephalopathy, or intensive care unit delirium (9, 10). The reductionist approach of evaluating single candidate molecules, while methodologically straightforward, appears inadequate for a disorder driven by perturbations across multiple molecular networks rather than isolated pathway abnormalities (10).

Within the framework of systems immunology, multi-omics technologies—including genomics, transcriptomics, proteomics, and metabolomics—represent a fundamentally novel approach to biological investigation. Rather than focusing on isolated molecular entities, these methodologies capture molecular data across multiple biological strata concurrently, thereby facilitating the systematic delineation of disease-associated networks rather than individual molecular components. Their utility in the study of SAE extends beyond mere data generation; they establish a technological foundation for molecular subtyping. Given that SAE likely encompasses multiple endotypes characterized by distinct pathogenic mechanisms, only a multidimensional molecular signature can accurately define these subgroups. Furthermore, the integration of multi-omic datasets enables the elucidation of cross-layer regulatory interactions—for instance, the influence of genetic variants on transcriptional activity, which subsequently modulates protein networks and metabolic pathways—phenomena that single-omic analyses are unable to detect (11). This systems-level perspective is critical for advancing beyond descriptive phenomenology toward mechanistic insights and, ultimately, the development of precision therapeutics.

In this review, we do not simply enumerate recent omics findings related to SAE. Instead, we pursue two primary objectives: first, to assess whether evidence from genomic, transcriptomic, proteomic, and metabolomic investigations converges upon shared pathological pathways, and if so, to identify the loci of such convergence; second, to delineate the principal obstacles impeding the translation of multi-omic discoveries into clinical applications and to propose systematic strategies to surmount these challenges. Drawing upon our synthesis, we introduce an integrated “three-tier cascade” conceptual model of SAE pathogenesis (presented as a heuristic framework rather than a proven temporal sequence, acknowledging that the temporal ordering of events remains unresolved and likely depends on SAE subtype and time course) and outline a methodological framework—spanning study design through clinical validation—intended to expedite biomarker qualification and therapeutic innovation (**Figure 1**). Our goal is to provide not only a comprehensive overview of recent multi-omics advancements in SAE but also a pragmatic roadmap to facilitate the transition from discovery to clinical implementation.

**Figure 1:**
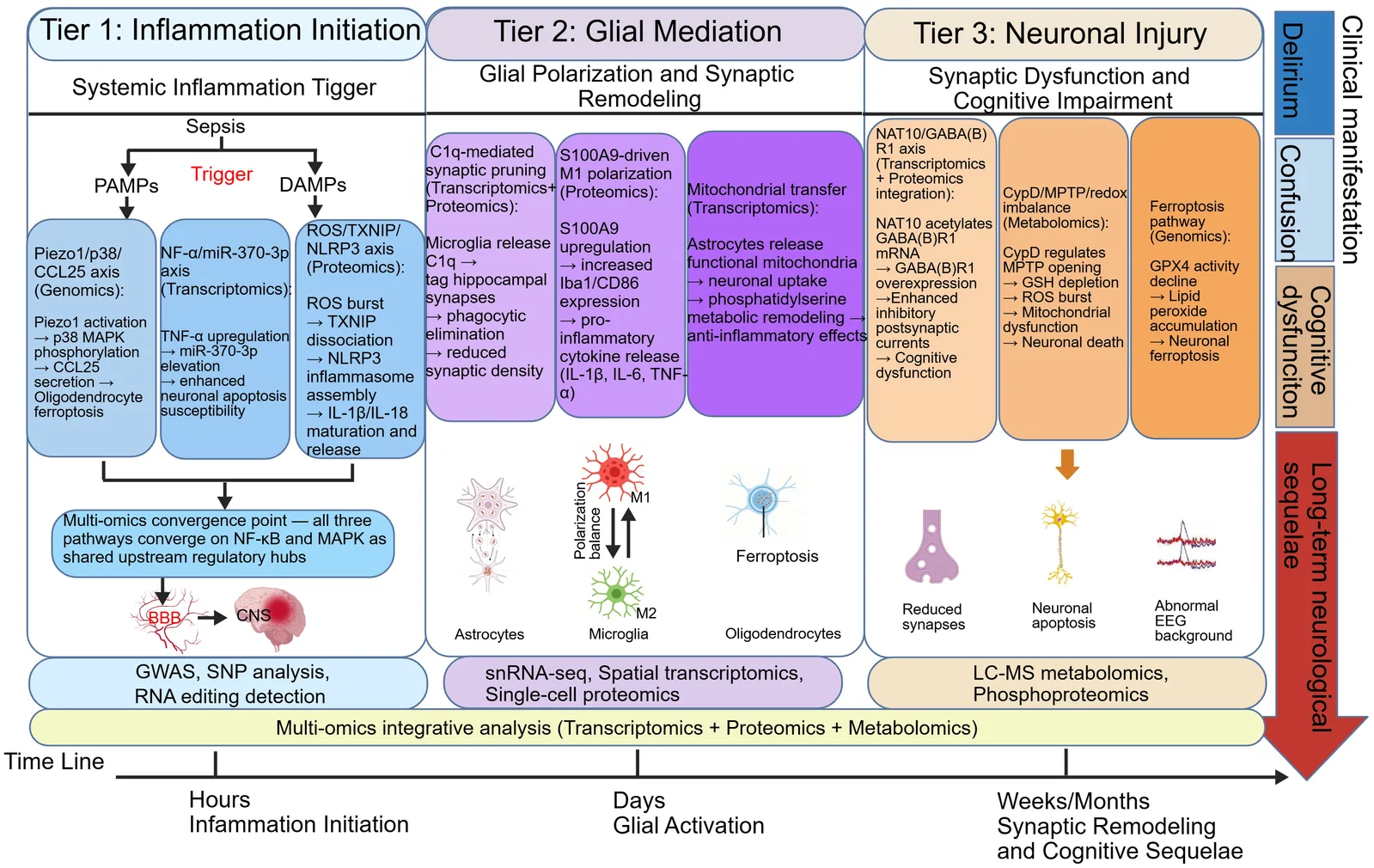
An integrated multi-omics conceptual model of the three-tiered cascade mechanism in sepsis-associated encephalopathy (SAE). This schematic integrates findings from genomics, transcriptomics, proteomics, metabolomics, and multi-omics integrative analyses into a hierarchical framework of SAE pathogenesis. Tier 1 (Systemic Inflammation Initiation): Peripheral inflammatory signals triggered by sepsis activate Piezo1/p38/CCL25 (genomics), TNF-α/miR-370-3p (transcriptomics), and ROS/TXNIP/NLRP3 (proteomics) pathways, which converge on NF-κB and MAPK as upstream signaling hubs. These cascades compromise the blood–brain barrier and transmit inflammatory signals to the central nervous system. Tier 2 (Glial Mediation): Microglia undergo pro-inflammatory and repair-associated states polarization, with S100A9 driving pro-inflammatory polarization (proteomics), while C1q-dependent synaptic pruning (transcriptomics and proteomics) eliminates hippocampal synapses. Astrocytes contribute through mitochondrial transfer to neurons (transcriptomics), modulating lipid metabolism and inflammatory responses. This tier represents the most densely corroborated layer of multi-omic evidence. Tier 3 (Neuronal Injury and Cognitive Impairment): Integrative transcriptomic–proteomic analysis reveals the NAT10/GABA(B)R1 axis mediating inhibitory synaptic dysfunction, while metabolomics identifies CypD/MPTP-driven redox imbalance and ferroptosis pathways contributing to neuronal death. Clinically, these molecular events translate to delirium, cognitive dysfunction, and long-term neurological sequelae. The bottom timeline indicates the temporal progression from hours (inflammation) to days (glial activation) to weeks/months (synaptic remodeling and cognitive sequelae), with corresponding omics technologies annotated at each stage. BBB, blood–brain barrier; DAMPs, damage-associated molecular patterns; GSH, glutathione; MPTP, mitochondrial permeability transition pore; ROS, reactive oxygen species; snRNA-seq, single-nucleus RNA sequencing. We created this image on the BioGDP.com website.

## 2 Omics studies in sepsis-associated encephalopathy

The subsequent four sections systematically review principal discoveries from genomics, transcriptomics, proteomics, and metabolomics in SAE research. Investigation depth varies considerably across these domains: transcriptomics—particularly non-coding RNAs and microglial profiling—has generated the most data, followed by proteomics and metabolomics, while genomics remains nascent. Technical principles were addressed in the Introduction and will not be reiterated. Instead, we address: What key molecular events has each omic layer identified? Do these findings cross-validate? Which molecules have advanced to interventional validation or clinical translation? Representative biomarkers and therapeutic targets are summarized in **Tables 1**, **2**.

**Table 1:** Potential biomarkers for SAE identified through multi-omics approaches.

Omics layer	Biomarker	Species/Model	Sample type	Key finding	Validation stage	Reference
Genomics	IL-8	Human	CSF	Key extracellular factor with diagnostic potential	Discovery	(12)
Genomics	Vegfa, Pecam1, Cdh5, Vwf, Fcgr3, C1qa/b/c, Fcgr4, Fcgr2b	Mouse (bioinformatics)	Brain tissue	Therapeutic targets for preventing SAE progression	Discovery (integrative analysis)	(13)
Genomics	gpr4, SOCS3, BAG3, ZFP36, CDKN1A, ADAMTS9, gadd45b	Human (bioinformatics)	Bioinformatics	Diagnostic value in SAE and hepatic encephalopathy	Discovery (WGCNA)	(8)
Transcriptomics	miR-370-3p	Mouse/Human	Plasma, brain tissue	TNF-α-induced neuronal apoptosis; SAE biomarker	Discovery (cohort validation)	(14)
Transcriptomics	miR-29a	Human	Peripheral blood	Correlates with NSE, S100β, IL-6; predicts SAE and poor prognosis	Discovery (case-control)	(15)
Transcriptomics	miR-127-3p, miR-423-3p, miR-378b, miR-106-3p	Rat	Exosomes (brain, CSF, plasma)	Differential expression across biofluids	Discovery (exploratory)	(16)
Transcriptomics	S100a8, Upp1, Mt2a	Rat (young vs. aged)	Brain tissue	Increased in young septic rats; age-related response	Discovery	(17)
Proteomics	PON1, THBS1, FGG (up); IgHV3, C1QC (down)	Human	Plasma exosomes	Immune regulation, coagulation, lipid metabolism alterations in SAD	Discovery (pilot, n=small)	(18)
Proteomics	SMAD4, DPYS, PTGDS (down); CUL4A (up)	Mouse	Brain tissue	Associated with 2% hydrogen gas protective mechanism	Discovery (iTRAQ + WB)	(19)
Metabolomics	4-hydroxyphenylacetic acid	Human	Plasma	Correlates with consciousness impairment severity	Discovery (GC-MS)	(20)
Metabolomics	Malic acid, oxalic acid	Human	Plasma	Common differential metabolites in SAE	Discovery	(20)

**Table 2:** Therapeutic targets for SAE identified through multi-omics approaches.

Target	Omics layer	Species/Model	Specimen/Sample type	Therapeutic intervention	Mechanism	Development stage	Reference
Piezo1	Genomics	Mouse (Piezo-KO)	Brain tissue/microglia	siRNA targeting Piezo1	Inhibits p38 pathway, reduces CCL25/IL-18, prevents oligodendrocyte ferroptosis	Preclinical (*in vivo*)	(21)
ICAM-1, IRF7, IL-1β, CCL2, IL-6, SOCS3	Transcriptomics	Mouse	Brain tissue	Ulinastatin	Potential therapeutic targets identified through transcriptomic sequencing	Preclinical (transcriptomics)	(22)
C1q complement	Transcriptomics	Mouse/Human	Hippocampus/plasma	C1q blocking antibody, CSF1-R inhibitor	Inhibits microglial C1q-tagged synaptic pruning; protects neurons and improves cognition	Preclinical (*in vivo*)	(23)
PI3K/AKT/Nrf2	Transcriptomics	Mouse	Brain tissue	Cuscuta chinensis flavonoids (CCLF)	Anti-apoptotic, anti-inflammatory, antioxidant effects	Preclinical (*in vivo*)	(24)
PPAR-γ/TLR4/CXCL10	Transcriptomics	Mouse	Brain tissue	Pioglitazone	Improves SAE injury through inflammatory pathway modulation	Preclinical (*in vivo*)	(25)
ROS/TXNIP/NLRP3	Proteomics	Mouse (BV-2 cells)	Cell lysate	N-acetyl-L-cysteine (NAC)	Reduces ROS production, inhibits NLRP3 inflammasome activation	*In vitro*	(26)
SMAD4, DPYS, PTGDS, CUL4A	Proteomics	Mouse	Brain tissue	2% Hydrogen gas inhalation	Modulates protein expression to reduce SAE incidence	Preclinical (*in vivo*)	(19)
Nrf2/GPX4	Proteomics	Mouse		Irisin	Inhibits hippocampal ferroptosis; improves inflammatory microenvironment	Preclinical (*in vivo*)	(27)
Rras, RAS/MAPK	Proteomics	Mouse	Brain tissue	Orexin-A (OXA)	Inhibits MAPK pathway activation	Preclinical (*in vivo*)	(28)
NAT10/GABA(B)R1	Integrative (Transcriptomics + Proteomics)	Mouse	Hippocampal neurons	PLX3397, NAT10 inhibition	Reduces GABA(B)R1 expression, weakens inhibitory postsynaptic currents, improves cognition	Preclinical (*in vivo* +KO)	(5)
NLRP3 inflammasome	Integrative	Mouse	Brain tissue/gut microbiota	NU9056 (KAT5 inhibitor)	Inhibits NLRP3 activation; affects gut microbiota and metabolites	Preclinical (*in vivo*)	(29)
Notch1/NF-κB	Integrative	Mouse	Brain tissue/gut	Palmatine	Enhances neurological function; repairs intestinal barrier; reduces neuroinflammation	Preclinical (*in vivo*)	(30)
S100A9/M1 polarization	Integrative	Mouse	Hippocampus	Paquinimod	Promotes M2 polarization; improves survival and learning/memory	Preclinical (*in vivo*)	(6)
ROCK/PTEN/Akt/mTOR	Integrative	Mouse (HT22 cells)	Cell lysate/exosomes	M2 microglia-derived exosomes (miR-124-3p)	Inhibits neuronal apoptosis, reduces ROS, restores mitochondrial function	*In vitro*	(2)

### 2.1 Genomics

Genomics studies genome architecture, variation, editing, and function using whole-genome sequencing, DNA microarrays, and SNP analysis (31). In sepsis-associated encephalopathy (SAE), genomic research is less developed than transcriptomics and proteomics but offers unique value in characterizing genetic determinants of disease susceptibility—how genetic background influences vulnerability to sepsis-induced brain injury—and identifying genetic markers for risk stratification.

#### 2.1.1 Susceptibility genes and genetic variants

Through database mining and bioinformatic approaches, IL-8 was identified as a key extracellular factor in SAE CSF with diagnostic and therapeutic potential (human CSF, discovery cohort) (32). Zhang et al., integrating multiple datasets, found persistent microglial–neuronal interactions and identified Vegfa, Pecam1, Cdh5, Vwf, Fcgr3, C1qa/b/c, Fcgr4, and Fcgr2b as therapeutic targets for preventing SAE progression (mouse brain tissue, bioinformatic integration) (33). The p38 pathway is a member of the MAPK family; both are implicated in the same intercellular network, suggesting the MAPK cascade—including p38—represents a central node in SAE neuroinflammation.

Genome-wide association studies (GWAS) have expanded genetic variant discovery. In Piezo1-knockout mice, siRNA targeting Piezo1 suppressed CCL25/IL-18 via p38 inhibition, regulating the CCL25/GRP78 axis and preventing oligodendrocyte ferroptosis (mouse Piezo1-KO model, brain tissue) (27). SNP analyses revealed correlations between mitochondrial DNA haplogroups and delirium susceptibility in Caucasian and African American populations, though mechanisms remain to be fully clarified (human blood DNA, n=large cohort) (7).

In a comparative SAE–hepatic encephalopathy study, weighted gene co-expression network analysis (WGCNA) identified seven hub genes (gpr4, SOCS3, BAG3, ZFP36, CDKN1A, ADAMTS9, gadd45b) with diagnostic value in septic cirrhotic patients, with shared downstream pathways enriched in hypoxia, inflammation, and immune responses (human bioinformatic analysis, blood/clinical data) (34).

Collectively, these findings support the potential of miRNAs as SAE biomarkers, but translation requires prospective validation, standardized assays, and larger multi-center cohorts to confirm clinical utility.

#### 2.1.2 Epitranscriptomics: A-to-I RNA editing

A-to-I RNA editing, mediated by ADAR enzymes, plays regulatory roles in inflammatory and neurological diseases (35, 36). Zhang et al. found significantly reduced editing sites and edited genes in postmortem brain tissue from septic patients, correlating with gene expression changes (human postmortem brain tissue, n=8 sepsis cases) (29). Li et al. revealed time-dependent A-to-I RNA editing dynamics in cerebrovascular endothelial cells and microglia during SAE neuroinflammation (mouse LPS model, brain tissue) (58).

#### 2.1.3 Therapeutic targets identified through genomic and transcriptomic approaches

Genomic studies have provided preliminary evidence and candidate targets for therapeutic strategies in SAE, though these remain largely at the preclinical stage. Transcriptomic analyses of ulinastatin treatment identified ICAM-1, IRF7, IL-1β, CCL2, IL-6, and SOCS3 as potential therapeutic targets (mouse CLP model, brain tissue) (37). Although initially identified via transcriptomics, these targets bridge genomic and transcriptomic discovery as their genomic characterization is ongoing, and we include them here as examples of targets that bridge genomic and transcriptomic discovery.

#### 2.1.4 Current limitations

The application of genomics to SAE research faces several challenges. SAE genetic research largely focuses on common SNPs, with rare variants, structural variations, and CNVs largely unexplored. Besides, validation of promising targets such as Piezo1 is limited to single animal models and specific genetic backgrounds—specifically, C57BL/6 mice with a specific knockout; replication across strains, species, and non-genetic models is lacking. Third, most GWAS-identified loci reside in non-coding regions, and their downstream biological effects remain to be determined. Strategies to address these limitations are discussed in Section 4.3 and **Box 1**.

Box 1 **Methodological recommendations for future SAE multi-omics research.**

Five Guiding Principles

Principle Core Message

(1) Dynamic over static Longitudinal sampling captures disease trajectories; single timepoints cannot distinguish early signals from late sequelae.

(2) Patient-centered over model-centered Human specimens and real-world heterogeneity must be prioritized; animal findings require human validation.

(3) Multi-model over single-model Cross-model replication (LPS, CLP, pneumonia models) is the minimum bar for translational credibility.

(4) Causal over correlational Move beyond pathway enrichment toward causal inference (Mendelian randomization, Bayesian networks).

(5) Clinical utility over statistical significance Design all discovery with the goal of improving patient care, not merely achieving p < 0.05.

Operational Recommendations by Phase

Phase 1 – Study Design

· Three-timepoint longitudinal sampling (sepsis admission, SAE onset, recovery/sequelae), with each patient as their own control.

· Clinical phenotyping by delirium subtype, severity, temporal pattern, and outcome trajectory.

· Confounding control: age, baseline cognition, APACHE II/SOFA, cumulative sedative dose.

· Control groups: septic without encephalopathy, non-septic ICU with similar sedation, delirium from other etiologies.

Phase 2 – Model Systems

· Multi-model cross-validation (LPS + CLP + pneumonia models); validation = consistent changes across ≥2 models.

· Aged and comorbidity-bearing animals (≥18 months; ApoE^-^/^-^, db/db, metabolic syndrome) as mandatory, not exceptional.

· Humanized models: iPSC-derived tri-cultures, organ-on-chip, or xenotransplantation to bridge species gaps.

· Standardized phenotyping per MIASS guidelines (ligation length, puncture number, antimicrobial timing, fluid protocols).

Phase 3 – Data Integration

· Causal inference: WGCNA, network centrality, Bayesian networks, SEM, or Mendelian randomization.

· Individual-level integration: MOFA, iCluster, or similarity network fusion to capture patient heterogeneity (replace group averages).

· Composite risk scores using machine learning (LASSO, random forest, XGBoost) with nested cross-validation and external validation.

· Multi-organ biobanking: paired CSF, plasma, urine, feces, and multi-organ transcriptomics in animals (gut–brain, lung–brain, liver–brain crosstalk).

Phase 4 – Clinical Validation

Four-stage framework (all candidates should report their current stage):

Stage Description Criteria

1 – Discovery Single cohort Significant p-value

2 – Analytical validation Assay reproducibility CV < 15%

3 – Clinical validation Independent cohort AUC with 95% CI

4 – Clinical utility Prospective impact Randomized or longitudinal study

Currently, nearly all SAE candidates remain at Stage 1.

Additional recommendations:

· Multi-center biobanking consortia with harmonized protocols and adequate power (n ≥ 200 per group).

· Dynamic risk prediction using serial biospecimens and joint modeling (superior to single timepoints).

· Biomarker-guided basket/adaptive platform trials: stratify by molecular endotype, randomize to mechanism-matched interventions (anti-inflammatory for immune-dominant, mitochondrial protectants for metabolic-dominant, anti-ferroptotic for oxidative-dominant endotypes), and test treatment-by-endotype interactions.

### 2.2 Transcriptomics

Transcriptomics analyzes all RNA transcripts—mRNA, miRNA, lncRNA, circRNA—in cells, tissues, or individuals. It is the most data-rich layer in SAE multi-omics, having characterized glial–neuronal spatiotemporal dynamics at single-cell resolution. The following subsections address miRNA biomarkers, lncRNA/circRNA, single-cell/spatial transcriptomics, glial dynamics, therapeutic targets, and limitations.

#### 2.2.1 MicroRNAs: peripheral biomarkers with greatest translational potential

miRNAs are 20–24 nucleotide non-coding RNAs that regulate gene expression post-transcriptionally. Their stability in blood and ability to cross the BBB make them attractive non-invasive biomarker candidates (22, 23).

Visitchanakun et al. found miR-370-3p upregulated in SAE mouse brain and validated this in patient plasma; TNF-α induces miR-370-3p, enhancing neuronal apoptosis susceptibility (mouse brain + human plasma, discovery cohort) (38). Guo et al. found miR-29a, NSE, S100β, and IL-6 elevated in SAE patient peripheral blood, with miR-29a correlating with these markers (human peripheral blood, n=SAE cases vs. non-SAE sepsis) (12). Xiao et al. identified 101 differentially expressed exosomal miRNAs in SAE rat brain, CSF, and plasma; four (miR-127-3p, miR-423-3p, miR-378b, miR-106-3p) showed consistent expression across all three biofluids (rat CLP model, exosomes from brain/CSF/plasma) (39).

Despite theoretical advantages, most miRNA studies are single-cohort retrospective analyses with sample sizes under 100; prospective validation in independent cohorts is absent. Optimal miRNA panels versus single miRNAs have not been systematically compared, nor have stability across storage/processing conditions been adequately tested in SAE samples. Multi-center standardized studies are the next essential step.

#### 2.2.2 Long non-coding RNAs and circular RNAs: emerging regulatory networks

lncRNAs (>200 nucleotides) and circRNAs participate in inflammatory and neurodegenerative processes via competitive endogenous RNA activity, transcriptional regulation, and epigenetic modification.

Sun et al. found lncRNA and mRNA differential expression in SAE rat brain correlated with microtubule assembly and polarization (rat CLP model, brain tissue) (25). Wen et al. identified novel mRNA, lncRNA, and circRNA candidates in LPS-stimulated BV-2 microglia (mouse BV-2 cell line, *in vitro* LPS stimulation) (19).

We acknowledge the LPS/BV-2 model is an acute *in vitro* system that does not capture chronic, progressive SAE; relevance to full SAE spectrum requires confirmation in more chronic models and human samples. Current lncRNA/circRNA research relies almost exclusively on bulk tissue sequencing, lacking single-cell validation.

#### 2.2.3 Single-cell and spatial transcriptomics: a panoramic view of cellular heterogeneity

Bulk RNA sequencing pools signals from multiple cell types, masking differential responses. snRNA-seq and spatial transcriptomics (Stereo-seq) are transforming this landscape.

Zhu et al., combining snRNA-seq and Stereo-seq in LPS-induced SAE mice, identified pathological niches of Astro-2, Micro-2, and Vas-1 cells with upregulated ligand–receptor pairs (mouse LPS model, brain tissue, snRNA-seq + Stereo-seq) (60). This provided one of the first spatial descriptions of SAE-associated molecular alterations—a preliminary framework—but generalization requires replication across models and human tissue.

Hamasaki et al. found S100a8, Upp1, and Mt2a increased in young septic rat brain, while aged rats showed altered olfactory perception networks (rat CLP model, young vs. aged, multiple brain regions) (13). snRNA-seq and spatial transcriptomics face cost and tissue processing barriers limiting human SAE application.

#### 2.2.4 Glial transcriptional dynamics: from complement-dependent synaptic pruning to microglial depletion

Glial cells—astrocytes, microglia, oligodendrocytes—maintain neuronal function and synaptic homeostasis (41). In SAE, glial activation and hippocampal neuron degeneration are central to cognitive dysfunction.

Chung et al. found upregulation of innate immune, complement, and lysosomal pathways in septic mouse hippocampus, correlating with neuronal/synaptic damage. Microglial C1q activation and C1q-mediated synaptic pruning were increased; C1q blockade or CSF1-R inhibition suppressed pruning, reduced C1q, protected neurons, and improved cognition (mouse CLP model + human postmortem validation, hippocampus) (42). CSF1-R is not entirely microglia-specific; inhibition may affect peripheral monocytes. Depletion timing relative to sepsis onset significantly alters outcomes. Microglia-replacement and conditional knockout approaches are needed.

Wei et al., combining bulk and single-cell RNA-seq, found microglial depletion suppressed RNA editing of Apobec1, Apobec3, B2m, Mier1, and lncRNAs (Meg3, Snhg11) (mouse CLP model, hippocampal tissue, bulk + snRNA-seq) (18).

Kan et al. demonstrated astrocyte-derived mitochondria transfer to LPS-exposed neurons, remodeling neuronal transcript profiles (mitochondrial metabolism, inflammation, apoptosis) and lipid composition (increased phosphatidylserine) (mouse primary cell culture, astrocyte–neuron co-culture) (43).

#### 2.2.5 Transcriptomics-guided interventional studies

Boxiang et al. found Cuscuta chinensis flavonoids (CCLF) reduced inflammatory (TNF-α, IL-2, IL-6) and oxidative (MDA↓, SOD↑) stress, inhibited apoptosis, and reversed SAE hippocampal transcriptomic alterations via PI3K/AKT/Nrf2 (mouse LPS model, brain tissue) (44). Zhang et al. identified 620 DEGs in pioglitazone-treated SAE mice, enriched in inflammatory and chemokine pathways (mouse LPS model, brain tissue) (59).

Koutroulis et al. used mesenchymal stem cell-derived small extracellular vesicles (EV) in SAE mice, demonstrating transcriptomic restoration of aerobic metabolism and reduced pro-inflammatory cytokine expression (mouse CLP model, cerebellum transcriptomics) (45).

#### 2.2.6 Current limitations

Transcriptomics faces three challenges: (1) bulk RNA sequencing pools signals from multiple cell types; snRNA-seq partially addresses this but is limited by cost and sample availability; (2) thousands of DEGs are identified but no standardized method discerns functionally pivotal drivers from passengers; (3) transcriptomic biomarkers (e.g., miR-370-3p) remain at single-center retrospective validation without cross-center standardization. Strategies in Section 4.3 and **Box 1**.

### 2.3 Proteomics

Proteomics analyzes protein expression, PTMs, localization, and interactions. Compared to genomics/transcriptomics, it more directly reflects functional state; compared to immunoassays, it offers higher throughput, sensitivity, and specificity for novel marker discovery (24). In SAE, proteomics has validated inflammatory/oxidative pathways at the protein level, revealed energy metabolism and coagulation–immune crosstalk, and played a unique role in therapeutic mechanism characterization.

#### 2.3.1 Early discoveries and pathway validation

In 2007, Hinkelbein et al. performed the first SAE brain proteomics (2D electrophoresis + MS + IPA), identifying 24 proteins downregulated at 24h and metabolic/signaling protein changes at 48h (rat CLP model, brain tissue homogenate) (21). Yang et al., using iTRAQ-LC-MS/MS, quantified 3,163 proteins (57 up, 38 down) in septic rat brain, implicating microtubule metabolism, energy production, and neurological disease pathways (rat CLP model, brain tissue, iTRAQ-LC-MS/MS) (5).

#### 2.3.2 Protein-level evidence for inflammatory, oxidative stress, and NLRP3 inflammasome pathways

Zhao et al. validated LPS-induced BV-2 microglial responses: IBA1/CD68 upregulation, ROS production, NLRP3 activation; NAC partially reversed these via ROS/TXNIP downregulation (mouse BV-2 cell line, *in vitro*) (46). Antibody microarray studies showed severe sepsis cytokine mixtures promote PMN adhesion to cerebrovascular endothelial cells via β2-integrin–ICAM-1 (*in vitro* adhesion assay) (46). Liang et al. constructed a sepsis-specific serum proteomic co-expression network, identifying modules in lipoprotein remodeling, inflammation, and wound healing (human serum, sepsis cohort) (14).

#### 2.3.3 Protein markers from clinical specimens

Plaschke et al., using plasma exosomes from SAD patients, found upregulated PON1, THBS1, FGG and downregulated IgHV3, C1QC, enriched in immune regulation, coagulation, and lipid metabolism (human plasma exosomes, n=small pilot cohort) (47). This is one of few human CSF/blood SAE proteomic studies, but limited by small sample size and lack of independent validation.

#### 2.3.4 Proteomics-guided therapeutic mechanism elucidation

Xie et al. used iTRAQ-LC-MS/MS to identify 39 proteins associated with 2% hydrogen gas protection in septic mice; validation confirmed SMAD4, DPYS, PTGDS downregulation and CUL4A upregulation (mouse CLP model, brain tissue, iTRAQ + WB) (28). Subsequent phosphoproteomics revealed phosphoprotein and phosphorylation-mediated network alterations (mouse CLP model, brain tissue, phosphoproteomics) (48). Wang et al. found irisin inhibits hippocampal ferroptosis via Nrf2/GPX4 (mouse CLP model, hippocampus) (26). Guo et al. found intranasal orexin-A downregulated Rras/RAS and inhibited MAPK (mouse CLP model, brain tissue) (49).

#### 2.3.5 Current limitations

Three bottlenecks: (1) cross-specimen heterogeneity (postmortem brain, animal homogenates, CSF, blood differ considerably; no systematic comparison exists); (2) mass spectrometry platforms and analysis protocols lack standardization, preventing cross-study replication; (3) PTMs (phosphorylation, glycosylation, oxidative modifications) may be more pathologically relevant than abundance alone, but phosphoproteomics is scarcely applied (except hydrogen studies) and other PTMs unexplored. Strategies in Section 4.3 and Box.

### 2.4 Metabolomics

Metabolomics profiles small-molecule metabolites (<1000 Da) under defined conditions. As metabolites are terminal outputs of biological information flow, metabolomics is closest to the phenotypic endpoint. In SAE, it captures cerebral energy crisis, redox imbalance, and neurotransmitter abnormalities, with advantages for clinical translation due to stable peripheral detection. Platforms include NMR, LC-MS/MS, and GC-MS.

#### 2.4.1 Differential metabolite screening and diagnostic/prognostic markers

Zhu et al., using GC-MS, identified 63 differential plasma metabolites in SAE patients; four were most common: 4-hydroxyphenylacetic acid, 3-ethyl-4,7-dimethoxycarbostyril (35.8%), malic acid, and oxalic acid. 4-hydroxyphenylacetic acid correlated with consciousness impairment severity (human plasma, GC-MS) (50). This terminal phenylalanine/tyrosine metabolite may reflect both monoamine neurotransmitter and gut microbiota alterations—a hypothesis requiring multi-omics validation.

#### 2.4.2 Mitochondrial metabolic dysfunction and redox imbalance

MPTP opening collapses membrane potential, arrests ATP synthesis, and releases pro-apoptotic factors—central to metabolism, energy production, and cell death. Kobayashi et al. found CypD-KO mice maintained GSH levels, indicating CypD deficiency protects mitochondria by inhibiting excessive MPTP opening and reducing RO (mouse CLP model, CypD-KO vs. WT, brain metabolomics) (51). Hara et al. found edaravone improved 18h survival and reduced cortical/hippocampal neuronal death; metabolomics revealed CLP-induced sepsis decreased GSH/GSSG ratio and increased lipid peroxidation, reversed by edaravone (rat CLP model, brain tissue, CE-MS) (8).

#### 2.4.3 Metabolomics-guided interventional strategies

Using LC-MS/MS, Hara et al. evaluated a biomimetic antioxidant nano-formulation (mAOI NPs, PLGA encapsulating ROS-scavenging agent) in SAE mice; metabolomic data showed normalization of ceramides and phosphatidylcholines, suggesting lipid metabolic reprogramming underlies neuroprotection (rat CLP model, brain tissue, LC-MS/MS) (8). Xu et al., integrating transcriptomics and LC-MS metabolomics, identified 81 differential metabolites and 1,747 DEGs in SAE hippocampus (mouse CLP model, hippocampus, RNA-seq + LC-MS) (20).

#### 2.4.4 Current limitations

Four limitations: (1) fewer than 10 original SAE metabolomics studies, mostly from one group, without independent validation; (2) samples are predominantly brain homogenates or plasma; CSF metabolomics is exceedingly rare despite being most direct CNS reflection; (3) structural identification and absolute quantification remain technical bottlenecks—most report only relative abundance without isotope-labeled internal standards; (4) identified metabolic differences are largely terminal “stress” phenomena rather than upstream drivers; distinguishing causal from concomitant requires causal inference integrating transcriptomic and proteomic data (Section 4.3, **Box 1**).

### 2.5 Animal models in SAE omics research

This subsection contextualizes omic findings rather than comprehensively reviewing SAE models. The LPS injection model (primarily mice/rats) induces acute endotoxemia but lacks polymicrobial complexity. The CLP model, also predominantly in rodents, better mimics polymicrobial progressive sepsis but shows high inter-laboratory variability. Pneumonia-derived sepsis models (intratracheal LPS or live bacteria) are less common in omic studies but may better represent the most frequent clinical sepsis source.

## 3 Integrative multi-omics in sepsis-associated encephalopathy

The pathological essence of SAE is not a linear abnormality of a single molecular pathway, but a complex systemic disease in which genomic variation, transcriptional reprogramming, protein network perturbation, and metabolic remodeling are temporally and spatially coupled. Single-omic analysis captures only one cross-section of this system—genomics reveals “what could happen,” transcriptomics indicates “what is preparing to happen,” proteomics shows “what is happening,” and metabolomics outputs “what has already happened.” This hierarchical complementarity dictates that only through multi-omic integration can fragmented evidence be assembled into a complete pathological picture.

It is important to distinguish three levels of integration in the current literature: (a) parallel single-omic studies with pathway-level overlap (the majority); (b) true cross-omic integration where data from multiple layers are jointly modeled (rare); and (c) integration with causal validation, where multi-omic findings are corroborated by functional experiments (essentially only 52). This review adopts this classification throughout Section 3.

The following subsections address five themes—early integration efforts, transcriptomic–metabolomic coupling, transcriptomic–proteomic causal chains, glial–exosome-mediated intercellular regulation, and the microbiota–gut–brain axis—concluding with common methodological limitations.

### 3.1 Early integration efforts

In 2007, Hinkelbein et al. used two-dimensional gel electrophoresis and mass spectrometry to identify differentially expressed proteins in septic rat brain, followed by Ingenuity Pathways Analysis (IPA), identifying 11 of 35 regulatory proteins involved in metabolism, signaling, and cellular stress (rat CLP model, brain tissue, proteomics + IPA) (21). Although this was a proteomic-only study with downstream pathway analysis—not true multi-omic integration by current standards—it serves as a historical precedent for cross-layer interpretation from protein data to functional networks.

### 3.2 Transcriptomic–metabolomic coupling: a systematic map of hippocampal dysfunction

Xu et al., combining RNA-seq and LC-MS metabolomics in SAE mouse hippocampus, identified 1,747 DEGs and 81 differential metabolites. Pathway-level integration revealed coordinated dysregulation of lipid, amino acid, glucose, nucleotide metabolism, inflammation, and synaptic pathways (mouse CLP model, hippocampus, RNA-seq + LC-MS) (20). Metabolic changes were statistically coupled with inflammatory gene expression, indicating they are not independent but nested within the same regulatory network. This study represents one of the most systematic integrations reported, though it remains at pathway-overlap level—a limitation the authors acknowledge—and does not extend to causal inference.

### 3.3 Transcriptomic–proteomic causal chain: discovery of the NAT10/GABA(B)R1 signaling axis

Gao et al. established a complete causal chain from “RNA modification enzyme” to “receptor protein” to “synaptic function.” NAT10 promotes GABA(B)R1 expression via mRNA acetylation, enhancing inhibitory postsynaptic currents and causing cognitive dysfunction. Nat10 knockout or PLX3397 reduced GABA(B)R1 and improved cognition (mouse model, hippocampal neurons, transcriptomics + proteomics + KO + pharmacology) (52). This is the only SAE study combining cross-omic data with both genetic knockout and pharmacological causal validation—the methodological gold standard. We propose this as a template for future integrative studies.

### 3.4 Glial–exosome-mediated intercellular regulation

Microglia dynamically transition between pro-inflammatory and neuroprotective/repair-associated phenotypes (53), (17). Liao et al. found S100A9 inhibitor paquinimod suppressed S100A9 in SAE hippocampus, reduced pro-inflammatory markers, increased repair-associated markers, and improved cognition (mouse CLP model, hippocampus) (16). This established an “S100A9, pro-inflammatory polarization, cognitive impairment” chain but did not extend to transcriptional networks.

Exosomes (30–150 nm vesicles) carry proteins, RNAs, and lipids as intercellular mediators (54). Zhu et al. found M2 microglia-derived exosomes deliver miR-124-3p, regulating ROCK/PTEN/Akt/mTOR, inhibiting neuronal apoptosis, reducing ROS, and restoring mitochondrial function (mouse HT22 cells, exosomes, *in vitro*) (40). This study integrated exosomal miRNA (transcriptomics), protein phosphorylation (proteomics), and neuronal survival (phenotype)—a relatively complete cross-layer model.

Wei et al. found microglial homeostasis-dependent RNA editing changes correlated with lncRNA and immune gene expression alterations (mouse CLP model, hippocampal tissue, bulk + snRNA-seq) (18). Glial-exosome integration reveals cell-to-cell communication that neither transcriptomics nor proteomics alone could capture.

### 3.5 The microbiota–gut–brain axis: systemic integration through multi-omics

Recent years have seen growing interest in gut microbiota regulation of SAE. Chen et al. found KAT5 inhibitor NU9056 alleviated LPS-induced impairment via NLRP3 inhibition, with therapeutic effects correlated with gut microbiota restoration and altered metabolites (mouse LPS model, brain + gut microbiome + metabolomics) (55). Xu et al. found palmatine improved neurological function and reduced neuroinflammation via Notch1/NF-κB inhibition, integrating brain transcriptomics, gut 16S rRNA, and serum metabolomics (mouse LPS model, brain + microbiome + metabolomics) (56). In a systematic review, Zhang et al. consolidated the mechanistic basis of short-chain fatty acids (SCFAs) in SAE, proposing that SCFAs modulate neuroinflammation via the microbiota–gut–brain axis through enhancing intestinal barrier integrity, reducing systemic inflammation, and regulating microglial function (30). Liao et al. demonstrated that short-chain fatty acids protect cognitive function in SAE mice via G protein-coupled receptor 43 activation (mouse CLP model, brain tissue + feces, 16S rRNA sequencing +GC-MS) (15). Hu et al., in a prospective cohort study integrating 16S and ITS rRNA sequencing with fecal and plasma metabolomics, identified key gut bacteria, and microbiota-derived metabolites—particularly cysteinylglycine and tryptophan metabolic products—as critically involved in SAE pathogenesis, with multi-omics analysis revealing that gut microbiota-related tryptophan and glutathione metabolism pathways may play important roles in SAE development (human, rectal swabs + feces + plasma, 16S rRNA sequencing + ITS rRNA sequencing + untargeted metabolomics) (57).

Importantly, these gut-derived signals—SCFAs, bile acids, tryptophan metabolites—converge on the same three-tier cascade (Section 4.1): systemic inflammation (Tier 1), microglial polarization (Tier 2), and neuronal injury (Tier 3). The microbiota–gut–brain axis is thus not a separate pathway but an upstream modulator of this cascade.

### 3.6 Current limitations

Integrative multi-omics research in SAE remains nascent, with four common deficiencies: First, insufficient depth of integration—most studies are parallel single-omic analyses with pathway overlap, not systems biology integration. Second, lack of causal inference—except for NAT10/GABA(B)R1, few studies have established causal chains across omic layers. Third, population heterogeneity ignored—group-average integration may obscure subtype-specific signals. Fourth, no multi-organ integration—studies focus on brain or blood alone, missing inter-organ crosstalk. Strategies to address these limitations are discussed in Section 4.3 and **Box 1**.

## 4 From multi-omic discoveries to clinical translation: integration, limitations, and future pathways

### 4.1 An integrated conceptual model: the three-tier cascade of SAE pathogenesis

Synthesizing evidence across genomic, transcriptomic, proteomic, and metabolomic layers, the pathophysiology of SAE can be distilled into a three-tier cascade—inflammation initiation, glial mediation, and neuronal injury—with multi-omic data providing mutually reinforcing evidence at each level.

Tier 1 (Inflammation Initiation): Genomic studies have demonstrated that Piezo1 activation drives oligodendrocyte ferroptosis via the p38/CCL25 pathway (27); transcriptomic analyses have validated the TNF-α/miR-370-3p axis that enhances neuronal apoptosis susceptibility (38); and proteomic quantification has confirmed activation of the ROS/TXNIP/NLRP3 inflammasome cascade (46). Across all three layers, the evidence converges on early transmission of inflammatory signals from the periphery to the central nervous system as the triggering switch for SAE onset.

Tier 2 (Glial Mediation): This tier is the most densely corroborated by current multi-omic evidence. Single-cell transcriptomics has identified C1q-mediated microglial synaptic pruning as a critical mechanism of cognitive dysfunction (42), proteomics has further established that S100A9 upregulation drives microglial polarization toward the pro-inflammatory phenotype (16), and metabolomics has revealed that CypD-regulated mitochondrial metabolic reprogramming determines the intensity of glial inflammatory responses (51). These three lines of evidence converge firmly on the same conclusion: functional polarization of microglia constitutes the central hub of SAE progression.

Tier 3 (Neuronal Injury): Integrative transcriptomic and proteomic analyses have confirmed that the NAT10/GABA(B)R1 signaling axis mediates inhibitory synaptic dysfunction (52), while metabolomics has captured quantitative associations between metabolites such as 4-hydroxyphenylacetic acid and the severity of consciousness impairment (50).

We propose this three-tier cascade as a heuristic conceptual model to organize existing evidence, not as a proven temporal sequence. We acknowledge that the temporal ordering—whether inflammation always precedes glial activation, or whether mitochondrial dysfunction in neurons may occur simultaneously or even earlier—remains unresolved and likely depends on the SAE subtype and time course. The three tiers should be understood as interdependent domains rather than strict chronological phases. This model is intended to generate testable hypotheses, not to assert a completed mechanistic picture.

Importantly, these three tiers do not operate in linear sequence but form a positive feedback loop—damage-associated molecular patterns (DAMPs) released from injured neurons reciprocally activate glial cells, perpetuating a vicious cycle. This model suggests that combination strategies simultaneously targeting both inflammation initiation and the glial–neuronal interface are likely to be more promising than single-point interventions.

### 4.2 Critical contradictions and unresolved questions: intrinsic tensions in the current evidence base

The following critical contradictions—which we discuss in detail in Section 5—emerged from our synthesis and highlight the deeper challenges facing SAE research.

### 4.3 Bridging the translational gap: methodological pathways from discovery to validation

To address the above contradictions, we propose the following methodological restructurings (summarized in **Box 1**):

1. Shift study design from cross-sectional comparisons to longitudinal dynamic tracking. Future multi-omic studies should adopt a three-timepoint longitudinal sampling design—sepsis admission, SAE onset, and recovery/sequelae. Using each patient as their own control, this design can identify molecular signals marking the “qualitative transition point” from sepsis to SAE, while effectively eliminating inter-individual baseline variability and aligning more closely with real-world clinical monitoring needs.
2. Upgrade biomarker validation from single-cohort discovery to multi-center, stage-gated verification. Borrowing from the four-phase framework used in oncology biomarker development—discovery → analytical validation → clinical validation → clinical utility—SAE candidate biomarker studies should explicitly indicate their current phase. At present, the vast majority of candidates remain at Phase 1 (discovery), and the absence of subsequent validation is the core bottleneck in translational stagnation.
3. Expand model systems from single acute models to cross-model validation platforms. We recommend simultaneous use of the LPS model, CLP model, and pneumonia-derived sepsis model for cross-validation. Molecular changes that consistently replicate across multiple models carry substantially higher translational potential. Furthermore, aged animal models and comorbidity-bearing models (e.g., hypertension, diabetes) should become standard rather than exceptional.
4. Upgrade data integration from correlation to causation. Current multi-omic integration largely remains at the level of “transcriptomic + metabolomic pathway overlap analysis”—essentially a descriptive correlation. We recommend introducing Mendelian randomization, Bayesian network causal inference, and other such methods to move multi-omic data from “what correlates with what” to “what drives what” at the causal level.

The scarcity of human SAE multi-omic data reflects several converging obstacles: the difficulty of obtaining postmortem brain tissue with short autopsy intervals, the logistical challenges of serial CSF collection in unstable septic patients, and the lack of standardized biobanking protocols across ICUs. Additionally, the heterogeneity of SAE definitions across centers complicates harmonization. These barriers are not merely technical but also ethical and financial, requiring multi-center consortia and dedicated funding mechanisms to overcome.

Beyond animal models, patient-derived *in vitro* systems offer complementary translational platforms. iPSC-derived microglia–neuron–astrocyte tri-cultures exposed to serum or CSF from SAE patients can recapitulate patient-specific inflammatory responses. Brain organoids incorporating vascular components enable testing of blood–brain barrier integrity under septic conditions. CSF-derived exosomes from SAE patients provide a minimally invasive window into brain cell communication. These models are still emergent but hold promise for bridging the species gap and enabling personalized mechanistic studies.

### 4.4 The next frontier: technology-driven paradigm shifts

Looking ahead to the next five years, SAE multi-omic research will face transformative changes driven by technological innovation.

Spatial multi-omics will replace conventional tissue homogenate analysis, enabling *in situ* observation of gene expression and protein modification heterogeneity across different brain regions, cortical depths, and even single-cell spatial positions. This will be critical for understanding why SAE selectively damages specific regions such as the hippocampus.

Single-cell multi-omics (simultaneous transcriptomic, epigenomic, and proteomic profiling of the same cell) will resolve the current coarse conceptualization of “microglia” as a homogeneous population, revealing the truly pathogenic microglial subpopulations and their specific molecular signatures in SAE.

Organoid and microfluidic chip technologies hold promise for constructing *in vitro* “mini-brains” containing neurons, astrocytes, microglia, and vascular endothelial cells, allowing testing of multi-target combination interventions under controlled conditions and narrowing the species gap between rodents and humans.

The value of artificial intelligence and multimodal integration extends beyond high-dimensional data processing to its capacity for integrating non-omic data—jointly modeling multi-omic molecular profiles with clinical parameters (infection source, antibiotic regimens, sedation depth, imaging features). The output should not be a binary “SAE yes/no” classification, but rather a dynamic risk trajectory to guide real-time clinical decision-making.

Finally, we emphasize a fundamental reorientation: SAE is not a “genomic disease” or a “metabolic disease,” but rather the acute decompensation of the brain—a high-energy-demand organ—within a systemic illness. Future multi-omic research should therefore pay greater attention to the molecular mediators of inter-organ crosstalk (gut–brain, lung–brain, liver–brain axes), rather than exclusively focusing on molecular changes within brain tissue itself. Emerging evidence suggests that gut microbial metabolites (55) and liver-derived inflammatory mediators play significant roles in SAE, indicating that sample selection in multi-omic studies should expand from “brain tissue centrism” to multi-organ biospecimen linkage analysis.

In summary, multi-omic technologies have generated a sufficiently rich repository of molecular clues for SAE research. However, weaving these clues into an evidence network capable of guiding clinical decisions will require systematic upgrades across four dimensions: study design, validation strategy, model systems, and data integration methodology. Only through such a multi-dimensional advancement can SAE diagnosis move from “exclusion” to “precision assessment,” and treatment from “symptomatic support” to “targeted intervention.

## 5 Critical appraisal of the current evidence base: contradictions and unresolved questions

While multi-omic data have generated a wealth of findings, several important contradictions have emerged—and these tensions precisely delineate the deeper challenges facing SAE research.

### 5.1 Contradiction 1: the disconnect between molecular discovery abundance and clinical translatability

Dozens of candidate biomarkers have been reported (see **Table 1**), yet none has entered routine clinical testing. The root cause lies in the extreme-case contrasting design (severe SAE vs. healthy controls or non-SAE sepsis) that dominates most omic studies. While this design amplifies between-group differences, it sacrifices the clinically more critical threshold discrimination capacity—the ability to distinguish mild SAE from sepsis-associated clouding of consciousness, or to differentiate SAE from residual sedative effects—in everyday diagnostic scenarios.

### 5.2 Contradiction 2: the mismatch between reductionist mechanistic research and the systemic nature of SAE

Most studies focus on molecular changes in a single brain region (predominantly hippocampus) and a single cell type (predominantly microglia). However, the diverse clinical manifestations of SAE—ranging from delirium to coma—suggest involvement of functional decoupling across multiple brain networks. Recent spatial transcriptomic efforts (40) have begun to reveal pathological niche differences among cell subpopulations across brain regions, but these findings remain descriptive and have yet to reach the level of network-level mechanistic understanding.

### 5.3 Contradiction 3: the heterogeneity gap between animal models and clinical SAE

Approximately 90% of current studies employ acute LPS injection or CLP models. These models recapitulate systemic inflammation but fail to capture the real-world confounders of clinical sepsis—age, baseline cognitive status, pathogen diversity, antibiotic and sedative drug interactions—that profoundly influence outcomes. This explains why many interventions effective in animal models (e.g., hydrogen inhalation, irisin) have repeatedly failed in clinical trials: the omic data reflect model biology, not human patient biology.

### 5.4 Contradiction 4: the absence of standardized diagnostic criteria for SAE

The cited studies use variable definitions—Sepsis-3 plus CAM-ICU, Sepsis-3 plus EEG abnormalities, or autopsy-confirmed diagnoses—which complicates cross-study comparisons and may introduce selection bias. This fundamental issue is rarely discussed but underpins much of the heterogeneity in omic findings.

### 5.5 Contradiction 5: the predominance of cross-sectional sampling

Even the longitudinal recommendations in **Box 1** are forward-looking; currently, almost no multi-timepoint multi-omic datasets exist in SAE. This limits our ability to distinguish early predictive signals from late sequelae markers. The field urgently needs prospective longitudinal biospecimen collections.

### 5.6 Methodological implications

These five contradictions are not independent—they reinforce one another. The absence of standardized diagnostic criteria complicates cross-study comparisons (Contradiction 4), which in turn makes it difficult to assess whether findings from extreme-case designs (Contradiction 1) or single-model animal studies (Contradiction 3) are generalizable. The reductionist focus on single regions and cell types (Contradiction 2) is exacerbated by the lack of longitudinal data (Contradiction 5), as cross-sectional sampling cannot reveal whether observed molecular changes are causes or consequences. Breaking this cycle requires coordinated efforts across all five dimensions simultaneously—a point we return to in Section 4.3 and **Box 1**.

## Glossary

ADAR: Adenosine deaminase acting on RNA
A-to-I: Adenosine-to-inosine;
AUC: Area under the curve
BBB: Blood–brain barrier
CCLF: Cuscuta chinensis flavonoids
CI: Confidence interval
circRNA: Circular RNA
CLP: Cecal ligation and puncture
CSF: Cerebrospinal fluid
CSF1-R: Colony-stimulating factor 1 receptor
CypD: Cyclophilin D
DAMPs: Damage-associated molecular patterns
DEGs: Differentially expressed genes
DIA: Data-independent acquisition
EV: Extracellular vesicle
FDR: False discovery rate
FGG: Fibrinogen gamma chain
GABA(B)R1: Gamma-aminobutyric acid type B receptor subunit 1
GC-MS: Gas chromatography-mass spectrometry
GPX4: Glutathione peroxidase 4
GSH: Glutathione
GWAS: Genome-wide association study
ICAM-1: Intercellular adhesion molecule 1
IgHV3: Immunoglobulin heavy chain variable region 3
IL: Interleukin
IPA: Ingenuity Pathways Analysis
iPSC: Induced pluripotent stem cell
iTRAQ: Isobaric tags for relative and absolute quantitation
KAT5: Lysine acetyltransferase 5
KO: Knockout
LASSO: Least absolute shrinkage and selection operator
LC-MS/MS: Liquid chromatography-tandem mass spectrometry
lncRNA: Long non-coding RNA
LPS: Lipopolysaccharide
MAPK: Mitogen-activated protein kinase
MIASS: Minimum Information for Animal Sepsis Studies
miRNA: MicroRNA
MOFA: Multi-Omics Factor Analysis
MPTP: Mitochondrial permeability transition pore
mRNA: Messenger RNA
NAC: N-acetyl-L-cysteine
NAT10: N-acetyltransferase 10
NLRP3: NLRP3 inflammasome
NMR: Nuclear magnetic resonance
NSE: Neuron-specific enolase
OXA: Orexin-A
PON1: Paraoxonase-1
PTM: Post-translational modification
ROS: Reactive oxygen species
D: Sepsis associated delirium
SAE: Sepsis associated encephalopathy
SCFA: Short-chain fatty acid
SEM: Structural equation modeling
SNF: Similarity network fusion
SNP: Single nucleotide polymorphism;, snRNA-seq, Single-nucleus RNA sequencing
SOCS3: Suppressor of cytokine signaling 3
Stereo-seq: Spatial enhanced resolution omics sequencing
THBS1: Thrombospondin 1
TMT: Tandem mass tags
TNF-α: Tumor necrosis factor-alpha
TXNIP: Thioredoxin-interacting protein
WGCNA: Weighted gene co-expression network analysis
XGBoost: Extreme gradient boosting
