# Supplementary material for: Multi-omics advances in sepsis-associated encephalopathy: from pathogenesis to clinical translation
Source: Front Immunol. 2026 Oct 7;17:1941590. doi: 10.3389/fimmu.2026.1941590 (PMC13684868; doi:10.3389/fimmu.2026.1941590)
Supplement: Supplementary file 1 [file Table1.doc]

| **Study** | **Omic Layer** | **Sample Size** | **Species/Specimen** | **Statistical Correction for Multiple Comparisons** | **Independent Replication Cohort** | **Blinding of Outcome Assessment** | **Quality Summary** |
| --- | --- | --- | --- | --- | --- | --- | --- |
| **Hinkelbein et al., 2007** | Proteomics | n=10 rats per group | Rat brain homogenate | NR | No | NR | Exploratory; limited by technology of era; no correction or replication |
| **Sun et al., 2017** | Transcriptomics (lncRNA/mRNA) | n=6 rats per group | Rat brain tissue | NR | No | NR | Exploratory; small sample; no correction |
| **Visitchanakun et al., 2020** | Transcriptomics (miRNA) | Mouse: n=3/group; Human: n=26 SAE, n=18 control | Mouse brain; Human plasma | Yes (FDR) | No | NR | Mouse discovery + human validation; limited human sample size |
| **Guo et al., 2021** | Transcriptomics (miRNA) | n=42 SAE, n=40 non-SAE sepsis, n=40 healthy | Human peripheral blood | NR | No | NR | Moderate sample; case-control design; no correction reported |
| **Liang et al., 2021** | Proteomics | n=20 sepsis, n=20 control | Human serum | Yes (FDR) | No | NR | Moderate sample; correction applied; no replication |
| **Kobayashi et al., 2022** | Metabolomics | n=6–8 mice per group | Mouse brain tissue | NR | No | NR | Small sample; KO vs. WT comparison; no correction |
| **Xu et al., 2022** | Transcriptomics + Metabolomics (integrative) | n=6 mice per group | Mouse hippocampus | Yes (FDR for transcriptomics) | No | NR | Small sample; integration at pathway level; no replication |
| **Yang et al., 2023** | Proteomics | n=6 rats per group | Rat brain tissue | NR | No | NR | Small sample; iTRAQ discovery; no validation cohort |
| **Plaschke et al., 2023** | Proteomics (exosomes) | n=11 SAD, n=7 sepsis, n=7 control | Human plasma exosomes | NR | No | NR | Pilot study; very small sample; exploratory |
| **Chung et al., 2023** | Transcriptomics + functional | Mouse: n=5–8/group; Human: n=8 postmortem | Mouse hippocampus; Human postmortem brain | Yes | No | Yes (behavioral tests) | Mouse discovery + human validation; functional intervention; well-controlled |
| **Xiao et al., 2024** | Transcriptomics (exosomal miRNA) | n=6 rats per group | Rat brain, CSF, plasma exosomes | NR | No | NR | Small sample; exploratory; no correction |
| **Zhang et al., 2024** | Genomics (integrative bioinformatics) | Multiple public datasets (n varies) | Human/mouse brain tissue | Yes (FDR) | Yes (cross-dataset) | NA | Bioinformatics integration; cross-dataset validation; no experimental validation |
| **Li et al., 2024** | Genomics (WGCNA) | Multiple public datasets (n varies) | Human blood/tissue | Yes (FDR) | Yes (cross-dataset) | NA | Bioinformatics; cross-dataset validation; hub gene identification |
| **Gao et al., 2024** | Transcriptomics + Proteomics + functional | Mouse: n=5–10/group | Mouse hippocampal neurons | Yes (FDR) | No | Yes (behavioral tests) | Gold standard: multi-omic + KO + pharmacological validation; well-controlled |
| **Zhu et al., 2024** | Transcriptomics (snRNA-seq + spatial) | n=3 mice per group | Mouse brain (snRNA-seq + Stereo-seq) | Yes (FDR) | No | NR | No NR Cutting-edge technology; very small n; exploratory |
| **Kan et al., 2025** | Transcriptomics + Lipidomics | In vitro (n=3–6 replicates) | Mouse primary cell culture | NR | No | NR | In vitro; mechanistic focus; no replication |
| **Liao et al., 2022** | Metabolomics + Microbiome (SCFAs/GPR43) | Mouse: n=10 sham, n=15 CLP, n=15 CLP+SCFAs, n=15 | Mouse feces + brain tissue | NR | No | Yes (behavioral tests) | Mechanistic; GPR43 antagonist validation; multi-omics integration; well-controlled |
| Hu et al., 2026 | Multi-omics (16S rRNA + ITS rRNA + metabolomics) | Human: n=42 SAE, n=129 non-SAE sepsis, n=35 healthy controls | Human rectal swabs + feces + plasma | NR | No | NR | Discovery; prospective cohort with multi-omics integration; human cohort; no independent replication |

Supplementary Tabel 1. Methodological Quality Assessment of Key Multi-Omics Studies in SAE
